# Discriminating Spectral–Spatial Feature Extraction for Hyperspectral Image Classification: A Review

**DOI:** 10.3390/s24102987

**Published:** 2024-05-08

**Authors:** Ningyang Li, Zhaohui Wang, Faouzi Alaya Cheikh

**Affiliations:** 1 Faculty of Computer Science and Technology, Hainan University, Haikou 570228, China; ningyang799@gmail.com; 2 Faculty of Information Technology and Electrical Engineering, Norwegian University of Science and Technology, 2815 Gjøvik, Norway; faouzi.cheikh@ntnu.no

**Keywords:** hyperspectral image classification, discriminating spectral–spatial features, feature extraction, feature optimization

## Abstract

Hyperspectral images (HSIs) contain subtle spectral details and rich spatial contextures of land cover that benefit from developments in spectral imaging and space technology. The classification of HSIs, which aims to allocate an optimal label for each pixel, has broad prospects in the field of remote sensing. However, due to the redundancy between bands and complex spatial structures, the effectiveness of the shallow spectral–spatial features extracted by traditional machine-learning-based methods tends to be unsatisfying. Over recent decades, various methods based on deep learning in the field of computer vision have been proposed to allow for the discrimination of spectral–spatial representations for classification. In this article, the crucial factors to discriminate spectral–spatial features are systematically summarized from the perspectives of feature extraction and feature optimization. For feature extraction, techniques to ensure the discrimination of spectral features, spatial features, and spectral–spatial features are illustrated based on the characteristics of hyperspectral data and the architecture of models. For feature optimization, techniques to adjust the feature distances between classes in the classification space are introduced in detail. Finally, the characteristics and limitations of these techniques and future challenges in facilitating the discrimination of features for HSI classification are also discussed further.

## 1. Introduction

Hyperspectral imaging technology, which is actually a combination of imaging and spectrum techniques, can discover the characteristics hiding in many narrow bands of the reflected wave of a target [1]. Thanks to the enormous development of astronautical technology, a number of satellites with hyperspectral sensors have been launched over recent decades. From the early Terra [2], EO-1 [3], and HJ-1A [4] to the recent GF-5 [5], HysIS [6], PRISMA [7], EnMAP [8], etc., the spectral, spatial, and time resolutions of hyperspectral sensors have improved significantly. This enables hyperspectral remote-sensing images to present the subtle and rich information in visible and infrared wavelengths with hundreds of bands in a timely manner.

Classification, as one of the main missions of the analysis of hyperspectral images (HSIs) [9], aims to recognize the class of every pixel of a scenario using spectral and spatial information. These pixel-level classification results can be the basis of target detection [10], image fusion [11], and change detection [12]. Therefore, HSI classification has attracted broad attention in the fields of agriculture [13], urban planning [14], ecological management [15], the military [16], etc.

Unlike other types of images, HSIs consist of spectral and spatial dimensions simultaneously. On the one hand, each pixel is a one-dimensional (1D) spectrum that records the unique spectral reflective properties of land cover. On the other hand, each band is a two-dimensional (2D) image that describes the detailed spatial structures and positions of targets. From a holistic viewpoint, a spectrum and a band can be combined to gain joint spectral–spatial information. However, using the HSI cube/patch, which is composed of a center pixel and its neighborhoods, is a more general way to integrate the spectral and spatial dimensions. To express this inherent information sufficiently and effectively, a number of approaches, including traditional methods and deep-learning-based methods, have been proposed for HSI classification.

In the early days, traditional methods mainly focused on the extraction of hand-crafted features. For example, spectral amplitude [17,18,19] and gradient [20,21,22,23], as intuitive metrics, were exploited to characterize the spectral intensity and its variation. Histograms of oriented gradient (HOGs) [24,25,26,27] and local binary patterns (LBPs) [28,29,30] were effective tools to capture local shapes and textures. To extract joint spectral–spatial information, the Gabor filter [31,32,33], extended morphological profile (EMP) [34,35], and scale invariant feature transform (SIFT) descriptor [36,37,38,39] were implemented to describe the frequencies, structural relationships, and key elements hidden in HSI cubes or band-compressed images [40,41].

The aforementioned features were generally delivered to a classifier, such as a support vector machine (SVM) [23,34,42,43], *k*-nearest neighbor [44,45], linear discriminant analysis [46,47], etc., to seek out the optimal decision boundaries among different classes in high-dimensional classification space. To obtain the nonlinear representation, the kernel trick was introduced to transform data or features into the nonlinear or sparse space [48,49,50,51,52]. The performances of these methods are somewhat limited because those features are extracted by hand-crafted and fixed descriptors. The robustness of shallow representation is generally weak when the redundancy between bands is serious [53]. Extracting deep feature representation is an urgent demand in HSI classification.

Over the last decade, the constant evolution of graphical processing hardware has promoted the broad application of deep-learning techniques in the fields of computer vision [54,55] and natural language processing [56]. Compared with traditional machine-learning models, neural networks handle both feature extraction and classification in their entirety, which enhances the mutual optimization of parameters in both stages efficiently. Moreover, the fact that the layers of neural networks can be stacked to any depth makes the representation of deep features possible [57].

In order to classify HSIs, early deep-learning-based models, including artificial neural networks [58,59,60], deep belief networks (DBNs) [61,62], recurrent neural networks (RNNs) [63,64], long short-term memory (LSTM) networks [65,66,67], and auto-encoders (AEs) [68,69,70,71], mainly focused on the spectral features of each spectrum and the flattened spatial structures. Although the spectral dependencies were considered by RNN and LSTM models, the correlations between spectral and spatial information were often disregarded, making the classification accuracy unsatisfying.

To promote the completeness of spectral–spatial features, convolutional neural networks (CNNs) [54], which integrate filtration into the neural network, were introduced to HSI classification [65,72,73,74,75]. The flexible size of their filters enables CNNs to sense local spectral and spatial features on different scales with fewer parameters [73,76]. This joint spectral and spatial information [75] can be extracted by parallel 1D CNNs and 2D CNNs [77,78,79,80]. Different types of features [75,77,78] and probability scores [79,80] are fused for better classification results. Benefitting from cubic kernels, three-dimensional (3D) CNNs can directly extract the representative spectral–spatial features from HSI cubes and fuse them in each stage of the model [72,81,82,83,84,85,86]. Therefore, CNNs have been introduced to previous models to construct convolutional auto-encoders (ConvAEs) [87,88], convolutional recurrent neural networks (ConvRNNs) [89], convolutional long short-term memory (ConvLSTM) networks [90], graph convolutional networks (GCNs) [91], etc.

Certainly, there are some deficiencies in the architectures of CNNs. For instance, fully convolutional networks (FCNs) [92] solved the issue of fixed input size [93,94,95]. Capsule networks (CapsNets) [96] enhanced the ability of the model to sense the positional variances of objects [97,98,99]. The ghost module [100] removed redundant features with cheap linear transformations [101,102,103]. Deep convolutional generative adversarial networks (DCGANs) [104,105] generated more rational spectral signatures and spatial structures [106,107,108] to promote the convergences of CNNs.

The classification performances of the aforementioned deep architectures have been proved by many researches, but it is commonly difficult for deeper models to realize efficient convergence. This phenomenon caused by intervals between previous layers and posterior layers is called the vanishing gradient. It is often serious, especially for 3D CNN-based models. As the most appropriate solution, residual networks (ResNets) [109] connect previous layers and posterior layers with shortcut paths to ensure the effective back-propagation of gradients. ResNets were introduced to both spectral and spatial dimensions [110,111] to promote feature aggregation [112,113,114] and have been accepted by many backbones. In densely connected networks (DenseNets) [115], shortcut connections are also utilized between the current layer and rear layers to fuse the features at different levels and enhance feature complexity [116,117,118,119]. In summary, these methods have made incontestable progress in improving network architectures for feature extraction. To elevate the classification accuracy further, some special loss functions, such as contrastive loss [120], center loss [121], triple loss [122], and focal loss [123], have been utilized to adjust both the inter-class and intra-class distances of features [124,125,126,127,128,129,130] and enhance the importance of hard classes during network optimization [131,132,133,134,135].

Spectral–spatial features extracted by the above-mentioned algorithms are deep and complicated but may not be discriminating. An HSI cube contains the center pixel and its neighborhoods, which means that the interfering pixels with different labels from the center pixel may be also involved during feature extraction. Generally, these pixels will confuse the feature distributions of different classes due to their different spectral characteristics. In contrast, the relevant pixels having the same label as the center pixel will promote feature aggregation. Therefore, distinguishing both kinds of pixels is essential to represent the discrimination of spectral–spatial features for HSI classification.

To this end, a popular technique called the attention mechanism in the fields of neural machine translation [136,137,138] and computer vision [139,140,141,142] was introduced to capture salient spectral bands and relevant spatial areas of HSI cubes [143,144]. Many effective attention modules, such as self-attention (SA) modules [145], squeeze-and-excitation (SE) modules [146], convolutional block attention modules (CBAMs) [147], non-local modules [148], etc., have been proposed to enhance the discrimination of features. On the basis of the type of attention, these attention modules can be divided into the spectral and spatial attention modules.

Spectral attention modules aim to discover the salient bands of the target class. To reach this goal, SE modules [146] collect global semantic information [149,150,151] in spatial dimension with global average pooling layer and then map it to a special weight for each band with an MLP. The resultant spectral attention depends on global interactions between all bands, but local interactions may be neglected. To solve this problem, many extensions of the SE module, including efficient channel attention (ECA) modules [152,153,154], selective kernel (SK) modules [155,156,157,158], pyramid attention (PA) modules [159,160], spectral part of CBAMs (spe-CBAMs) [147,161,162,163,164], and frequency channel attention (FCA) modules [165,166,167,168], were designed to strengthen the relationships between various types of crucial information and spectral attention. This mode of global information transformation has been proven to be effective in locating the distinctive bands.

There are two patterns to capture relevant spatial areas in current spatial attention modules. The prototype of first pattern is the gather-and-excitation (GE) module [169]. It can gather spectral semantic representation within small regions and search for the responses between local representation and relevant areas [170,171,172]. Based on the GE module, the spatial part of CBAM (spa-CBAM) [147] introduced global max-pooling and large-scale convolution to capture the long-distance dependencies among pixels [161,162,173,174,175]. The bottleneck attention module (BAM) [176] adopted 1 × 1 × 1 convolution to elevate the nonlinear feature representation of attention [177,178,179]. But these modules are often affected by the interfering areas with a strong gray-value response.

The second pattern to acquire spatial attention is to measure the spectral correlations between pixels. The classic structures contain SA [145] and non-local [148] modules. They obtained the dot-product spectral similarity between all pixels for normalized weights [180,181,182,183]. They have been applied to highlight vital pixels in many architectures [184,185,186]. Considering the unique generation method of HSI cubes, a spectral-similarity-based spatial attention module (S^3^AM) [187] was designed to assess the spectral similarities between the center pixel and its neighborhoods adaptively. Compared with the former, the centralization ideology can highlight relevant areas and suppress interfering pixels to a great extent [150,188,189,190,191,192,193].

However, the above-mentioned spatial attention modules generally deduce a few modes of attention. To express possible spatial dependency sufficiently, transformers [194,195], which originate from the field of natural language processing and have been the core component of the ChatGPT model [196], adopt multi-head SA (MHSA) modules [181,197,198,199] to integrate various types of attention from different subspaces into a linear representation [200,201,202]. Transformer is also good at handling long-distance spectral dependency. But the use of the MHSA module results in considerable heavy time and computational costs for optimization. The swin transformer [203] adopts different sizes of shifted windows to explore local spatial relationships. Pyramid vision transformer [204] reduces the spatial resolution of attention to produce hierarchical fine-grained features. These transformer architectures handle the relevance between adjacent [205,206,207,208] and dense [209,210,211] pixels better and reach satisfying classification performances. Attention-mechanism-based models have almost been the predominant algorithms for HSI classification.

In summary, HSIs are provided with intricate spectral and spatial information, but the redundant bands and irrelevant areas may shrink the inter-class distance and expand the intra-class distance, which hampers algorithms to construct the discriminating feature distributions for classification to some degree. Therefore, researchers have paid increasing attention to these problems from traditional hand-crafted features to deep architectures and attention modules and proposed various effective methods and tricks to improve feature representation. In this article, techniques to discriminate features for HSI classification are summarized from the perspectives of feature extraction and feature optimization comprehensively. For feature extraction, including spectral features, spatial features, and spectral–spatial features, various techniques are summarized from the aspects of the characteristics of the data and the architectures of the models. For feature optimization, techniques to regulate the feature distribution in the classification space are illustrated. Moreover, the superiorities and limitations of the existing methods and possible future challenges to promote the discrimination of features for HSI classification are also pointed out.

The rest of this article is arranged as follows. Section 2 summarizes the techniques to promote the discrimination of features for HSI classification in detail. Section 3 analyzes some typical methods and points out their advantages and deficiencies. Future challenges are given in Section 4. Section 5 concludes this review.

## 2. HSI Classification Based on Discriminating Spectral–Spatial Features

Over the past decades, various algorithms based on machine learning and deep learning have been proposed for HSI classification. These algorithms generally possess two stages of feature extraction and classification. Feature extraction aims to represent the spectral features, spatial features, and spectral–spatial features of samples. Classification fits features of different categories into an appropriate distribution in decision space. Hence, the discrimination of features is the key to ensure a higher classification accuracy. To this end, many algorithms take inherent properties of hyperspectral data, network architecture, and feature optimization into full consideration.

**Figure 1:**
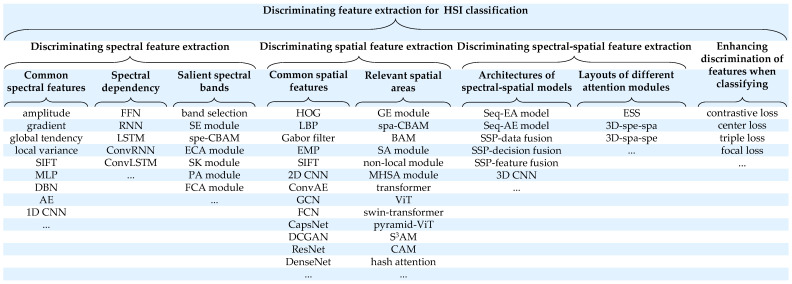
Overview of methods [17,20,24,28,31,34,36,38,39,58,61,63,65,67,68,70,72,74,76,77,81,89,90,91,92,96,106,109,115,119,124,126,128,131,145,146,147,148,150,152,155,159,164,165,169,176,187,189,190,197,200,202,206,209] pursuing the discriminating features for HSI classification.

As shown in Figure 1, techniques to represent the discriminating spectral features mainly focus on common features, spectral dependency, and salient spectral bands. Similarly, common spatial structures and relevant areas are valued for spatial feature extraction. Different architectures to combine spectral information and spatial information and handle the associations between salient bands and relevant areas are also essential to acquire the discriminating spectral–spatial features. During classification, some popular techniques to optimize the feature distances of hard classes are summarized.

### 2.1. Extraction of Discriminating Spectral Features

Spectral features, which are generally extracted from each spectra/pixels of HSI cubes, can be common features, such as spectral amplitude [17,18,19], spectral gradient [20,21,22,23], global tendency [36], etc. However, these manual features are generated by the feature descriptors designed empirically, which is not always effective and robust for complex HSI data sets. To improve the discrimination of spectral features, there are two important attributes worthy of attention, namely spectral dependency and the salient spectral band. Spectral dependency can be interpreted as a variety of correlations between bands. Salient spectral bands are a group of bands helpful for identification. Spectral features will be more representative if both attributes are considered during feature extraction. Next, techniques to develop common spectral features, spectral dependency, salient spectral bands are detailed.

#### 2.1.1. Common Spectral Features

##### Traditional Feature Descriptors

Spectral features are the implicit peculiarities and patterns of each spectrum of HSI and the foundation of classification. Shallow spectral features generally obtained by traditional feature descriptors include spectral amplitude [17,18,19], spectral gradient [20,21,22,23], global tendency [36,38], local variance [36,37,38,39], etc. Spectral amplitude is the original gray-scale value of a spectrum. Spectral gradient measures the variation in magnitudes in adjacent bands. However, both kinds of features are often interfered with by noise, which may deviate from decision results. To acquire stable spectral features, the SIFT descriptor was introduced to extract the overall tendency and local details from spectral curves. Global tendency is contextual information, while local details are marked variances. The comparison between this method and other spectral matching algorithms and minimum distance classifiers demonstrated the validity of 1D SIFT features [36,38,39]. However, the capability of the SIFT descriptor is still limited due to the fixed templates and complexity of HSIs.

##### Deep Network Architectures

Some classic techniques based on deep learning, including MLPs, DBNs, AEs, and 1D CNNs, have been favorable algorithms to gain deep and expressive nonlinear spectral features. MLPs [58,59,60] contain input layers, hidden layers, and output layers, which is the standard neural network architecture. The model receives the original spectrum as input and transforms the spectral features into nonlinear space with activation functions, such as rectified linear unit [212] and sigmoid. The classification results are then predicted by the output layer. Compared with traditional hand-crafted features, MLPs can generate abstract and diverse spectral features. To construct the joint probability distribution between data and label, DBNs which contain several restricted Boltzmann machines were applied to the process spectra of HSIs [61,62]. But a separate training mode limits the depth of DBNs, which may suppress the representation of local features. As a classic unsupervised learning model, AEs exploit the symmetrical pyramid structure to compress each spectrum into low-dimensional semantic information and relieve useless information [68,69,70,71]. These compressed features record the global trend of a spectrum and can be sent to various classifiers to predict possible labels. But due to the complete perception between spectrum and neuron, the ability of these models to explore local variances is often unsatisfying compared with CNNs [54]. Benefitting from variable 1D kernels, CNNs can capture both local variances and the global tendency of a spectrum [74,75,76,77,78,79,80]. The higher the number of convolutional kernels, the more types of features will be excavated. Different pooling modes can enhance the foreground and background information and reduce the spectral dimension. These advantages enable CNNs to extract complex features with fewer parameters.

#### 2.1.2. Spectral Dependency

Spectral dependency, which is caused by the reflective characteristics of objects and high spectral resolutions of HSIs, can be interpreted as the complex spectral relationship between bands. Making full use of spectral dependency is good for improving the discrimination of features and classification results because different types of land cover generally have unique reflective characteristics. The popular models to explore spectral dependency are mainly deep-learning-based models, such as feed forward networks (FFNs), RNNs, and LSTM networks.

FFNs, the normalized MLPs, depicted the global dependency between all bands with full connections [58,59]. But this property may be harmful to local dependency. As the classic model for natural language processing, RNNs were applied to capture the sequential correlations between neighboring bands [63,64]. Different from FFNs, the neurons of each layer are connected in RNNs. Each band of the spectrum is seen as a word of a sentence by RNNs. This structural consistence can discover the short dependency between neighboring bands for improving spectral feature representation. As an extension of RNNs, LSTM networks introduce memory units to replace regular neurons, which enhances the ability to sense the spectral dependency between farther bands [65,66,67]. They can acquire both long and short spectral dependencies. Furthermore, LSTM networks mitigate the issue of vanishing gradients of RNNs with a flexible gating mechanism. Moreover, the combinations of these models and CNNs, including ConvRNNs [89] and ConvLSTM networks [90], can extract an accurate sequential dependency. Therefore, RNNs and LSTM models have been the appropriate tools to represent complete spectral dependency and enhance the discrimination of spectral features.

#### 2.1.3. Salient Spectral Bands

Salient spectral bands are the exclusive and informative bands of HSIs. These bands may not possess strong reflective energy but are helpful to represent the distinguishing characteristics of a certain class. In other word, emphasizing salient bands makes more contributions to the extraction of discriminating spectral features.

##### Dimensionality Reduction and Band Selection

Band redundancy, caused generally by the similar reflectivity of objects in adjacent wavelengths, is a common factor to hinder the recognition of salient bands. Principal component analysis (PCA) [40,41,181] is a favorable method to alleviate this problem. It can unearth influential information by solving feature vectors. But the initial order of the bands is disturbed in the resultant components, which often abandon spectral dependency.

Another early method to reduce redundant information is band selection [81]. It selects a set of important bands based upon amount of information [213,214] and spectral similarity [215,216] for subsequent analyses. But these bands are chosen for all classes, which is a global optimization and may not be appropriate for each class.

##### Spectral Attention

Spectral attention which is a deep-learning-based algorithm aims to establish a connection between salient spectral bands and input. This advanced technique helps models to focus on different salient bands of different classes during feature extraction and classification. Many approaches have been used to receive effective spectral attention. The classic spectral attention module is the SE module [146]. It contains a global average pooling layer and a lightweight MLP. The former is exploited to gather global semantic information while the latter aims to compress and transform information to the importance of bands. As a plug-and-play module, SE modules can be embedded into anywhere in models to emphasize salient bands and elevate the discrimination of features [149,150,151].

The subsequent spectral attention modules, such as ECA modules [152], SK modules [155], PA modules [159], spe-CBAMs [147], and FCA modules [165], were mainly proposed to resolve the drawbacks of SE modules. ECA modules replaced the fully connected layer with a convolutional layer to facilitate the local interactions between bands [153,154]. SK modules and FCA modules enhanced the effectiveness of spectral attention with different scales of features [156,157,158] and frequency features [166,167,168], respectively. By introducing adaptive average pooling [160] and global max-pooling [161,162,163,164], PA modules and spe-CBAMs perceived different scales of contextual information and global salient responses, separately. These attention modules have become powerful means to capture salient bands for discriminating spectral features. However, almost all of these spectral attention modules cannot capture the multiple attention patterns which are important for extracting different semantic features of identical inputs.

### 2.2. Extraction of Discriminating Spatial Features

Spatial features include textures, edges, key points, shapes, etc., which can be extracted by traditional feature extractors [24,28,32,34,37] and deep neural networks [68,73,75,76,86], from each band of an HSI cube. But the discrimination of these features may be weak because relevant spatial areas tend to be ignored. Relevant spatial areas are mainly composed of the pixels which have the same label as the center pixel of the sample. Features extracted from these areas generally reveal the distinctive information of each class [162,171,178]. In this section, techniques to obtain common spatial features and relevant spatial areas are outlined.

#### 2.2.1. Common Spatial Features

##### Traditional Feature Descriptors

Spatial features can be interpreted as the spatial structures and relative positions of objects. They locate in each band which is actually a gray-scale image. Even though the spatial resolutions of hyperspectral sensors are generally lower than those of visual sensors, some algorithms used to analyze ordinary red–green–blue images can also be applied to extract the spatial features of HSIs. For example, HOGs were employed to acquire the regions with intense fluctuations of gray-scale values, including edges and corners [24,25,26,27]. LBPs were introduced to capture local invariant spatial textures [28,29,30]. SIFT descriptors were exploited to discover the stable structures composed of key pixels [36,37,38,39]. However, the spectral correlations of these spatial features cannot be found from separate bands. Due to the reflective discrepancy, the spatial structures in each band may be different. To improve the efficiency of feature extraction, PCA was utilized to extract the prime spatial information [40,41,86]. But this measure cannot take spectral correlation into account due to the loss of band order. Thus, the dimension of the Gabor filter [31] was extended to process both spatial and spectral domains [32,33,77]. Similarly, EMPs were applied to integrate the spatial structures of several bands, thereby enhancing local spectral correlations [34,35].

##### Deep Network Architectures

Because of the complexity of HSI, the robustness of these shallow spatial features is often not ideal. To extract abstract and deep spectral features, many algorithms based on deep learning have been proposed in recent years. CNNs [54], as one of the most popular models, can extract various local spatial features with different convolutional kernels and retain semantic and prominent spatial information with pooling operations [72,73,74,75,76,77,78,79,80,81,82,83,84,85,86]. Many previous models attempted integration with CNNs, such as ConvAEs [70,71,87], ConvRNNs [89], ConvLSTM networks [90], GCNs [91], and DCGANs [106,107,108], to supplement deep spatial feature representation for classification. In particular, FCNs [92] replaced classification layers with convolutional layers to allow varying sample sizes [93,94,95], which improves the portability of the trained model.

Generally speaking, the features in shallow layers include the aforementioned various general spatial structures while the features in deep layers gradually become more abstract and special. Nevertheless, deeper models cannot be optimized efficiently because the gradients of deep layers cannot be normally propagated to shallow layers. To cope with this deficiency, ResNet [109] was proposed to build the shortcuts between deep layers and shallow layers. These simple shortcuts allow gradients to flow between layers effectively. Thus, residual modules can be stacked at any depth to greatly improve the deep spatial features of HSIs [110,111,112,113,114]. This kind of trick was also reflected in DenseNets [115] and CapsNets [96]. DenseNets adopted the dense connection technique to aggregate different levels of spatial features and enrich feature complexity [116,117,118,119]. But this technique also leads to a heavy optimization burden. CapsNets replaced scalar neurons with vectors to discover the dynamical attributes of spatial features [97,98,99]. Compared with traditional feature descriptors, CNN-based algorithms, especially for ResNet, have been favorable ways to obtain deep spatial features.

#### 2.2.2. Relevant Spatial Areas

Spatial features extracted by deep models are the abstract integrated representation of high-level features. But these features may be not discriminating because these models generally treat each pixel fairly. In other word, some important pixels of a sample tend not to be valued during feature extraction. These pixels having the same class as the center pixel form relevant spatial areas. The spectral characteristics of pixels in these areas are similar and beneficial to feature aggregation [95,150,162,187]. On the contrary, other interfering pixels having different classes with the center pixel may introduce useless features. Therefore, it is necessary for current models to emphasize relevant spatial areas and suppress interfering spatial areas to ensure the discrimination of spatial features. To this end, various spatial attention modules were proposed to infer relevant areas [170,178,184,187,193,198,200,205,208]. According to the pattern of information processing, existing spatial attention modules can be divided into the convolution-based, similarity-based, and centralized spatial attention modules.

##### Convolution-Based Spatial Attention

Convolution-based spatial attention modules [170,173,175] usually adopt convolutional layers to connect the local correlations between regions with spatial attention. GE modules [169] utilize depth-wise convolution to gather and assess the correlations between spectral features in small regions and resize aggregated weights for adjustment [170,171,172]. To consider more useful information of input, spa-CBAMs [147] introduce global average pooling and max-pooling layers before convolution, which improve spatial attention without increasing the number of parameters [161,162,163,164,173,174,175]. Different from GE modules and CBAMs, 1 × 1 × 1 convolutional layers were exploited in BAMs [176] to compress and transform the information in spectral and channel dimensions [177,178,179], which enhanced the adaptation of spatial attention. Moreover, the three attention modules all applied the technique of the large scale of convolution, i.e., extended convolutions of GE modules, 7 × 7 convolutions of CBAMs, and dilated convolutions of BAMs, to cope with local spatial relationships better. However, this kind of attention module may be affected by interfering areas and cannot notice relevant areas far from the center pixel.

##### Similarity-Based Spatial Attention

Unlike the former models, similarity-based attention modules [150,187,188,190,191,193,200,205,211] measure the spectral similarity between pixels to decide the importance of each pixel. The classic SA modules [145], which were used to locate the crucial words of sentences in the field of neural machine translation, adopted the dot-product similarity to evaluate the spectral correlations between all pixels [180,181,182]. The generation of spatial attention was actually an operation on query, key, and value sets. The SA module has the power of capturing global dependency and partial local associations between pixels compared with convolution-based spatial attention modules. As an instance of an SA module, non-local networks [148] were introduced into the spatial, spectral, and channel dimensions to highlight more meaningful pixels, bands, and kernels [184,185,186].

The SA module is also one of the core components of transformer architectures [200,201,202]. A series of SA modules were integrated to construct MHSA modules [197,198,199], which can assist transformers to describe different modes of spatial attention in separate feature subspaces. However, the ability of the SA module to explore different scales of local spatial correlations is generally weak because its fully connected layers make it difficult to understand global spectral information. As an upgrade of the SA module, swin transformers [203] explored the correlations between different blocks with different sizes of shifted windows [205,206,207,208], and pyramid vision transformers [204] adjusted the resolutions of spatial attention and enriched feature hierarchy [209,210,211]. The transformer architecture has been the mainstream attention network for HSI classification but the optimization of it is usually time-consuming because of the complex high-dimensional matrices in SA modules. Another common deficiency which cannot be ignored is that the useless correlations between interfering pixels may be considered for the generation of spatial attention.

##### Centralization Ideology-Based Spatial Attention

To solve the deficiencies of SA modules, many algorithms based on centralized ideology, including S^3^AMs [187], spatial proximity modules [188], center attention modules (CAMs) [189], adaptive hash attention modules [190], etc., [191,192,193], have been proposed to emphasize the spatial structures related to the center pixel. Different from the SA module, this kind of attention module measures robust spectral similarity and activates the similarity adaptively. The similarities between the center pixel and others were retained merely for spatial attention. This kind of attention module can discover relevant areas precisely and reduce computational cost. In a word, the above-mentioned three types of spatial attention modules have made great progress in extensively capturing relevant areas for discriminating spatial features.

### 2.3. Extraction of Discriminating Spectral–Spatial Features

#### 2.3.1. Architectures of Spectral–Spatial Models

**Figure 2:**
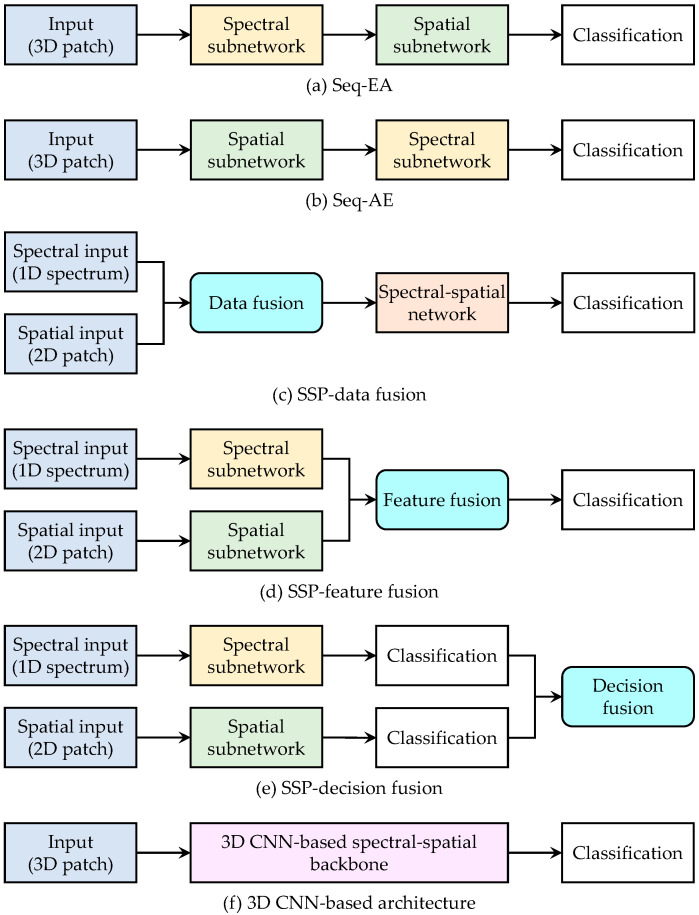
Architectures of six kinds of spectral–spatial models.

Spectral–spatial features are extracted by spectral–spatial models from both spectral and spatial dimensions of HSIs. As shown in Figure 2, spectral–spatial models generally adopt sequential, parallel, 3D architectures to integrate spectral and spatial information. This can be an influence factor of the discrimination of spectral–spatial features because different architectures may hamper the balance and relationships between spectral and spatial features. Similarly, the layout of spectral and spatial attention modules in models should also be taken into account to manage the importance of salient spectral bands and relevant spatial areas.

Spectral–spatial models are designed to obtain comprehensive features from both spectral and spatial dimensions of HSIs. In this section, considering that the features extracted by deep neural networks are often more complex than those of traditional hand-crafted features, spectral–spatial models based on deep learning are referenced. These models can be divided into four classes.

##### Sequential Spectral–Spatial (Seq-EA) Models

The first one arranges the spectral subnetwork and spatial subnetwork sequentially [76,86,88,95,97,209]. This kind of model usually collects global spectral information with 1D convolutional layers or RNNs and then exploits 2D convolutions to process spatial features for classification. Seq-EA models deem that spectral features can be embedded into each pixel and spatial structures are more significant for classification.

##### Sequential Spatial–Spectral (Seq-AE) Models

In contrast, the second one places the spatial subnetwork before the spectral subnetwork [63,65,67,154,210]. The spatial contextual information of samples were compressed into the spectral domain and 1D convolutions or RNNs were used to extract spectral features for classification. Compared with the first one, the architectures of Seq-AE models can be more lightweight.

##### Spectral–Spatial in Parallel (SSP) Models

Sequential models cannot take full advantage of spectral and spatial features for classification. The idea of integrating spectral and spatial subnetworks in parallel was found by the third kind of model. The inputs of them can be spectra and images or share the same HSI cubes. To improve classification performances, SSP models generally adopt the three modes of data fusion, decision fusion, and feature fusion to make full use of the spectral and spatial features.

The SSP models based on data fusion extract spectral–spatial features from the combination of spectral input and spatial input [61,62,69,75]. To implement this kind of model both spectral and spatial inputs are required to have an identical shape. More importantly, the following spectral–spatial networks should also be good at processing both spectral and spatial information simultaneously, which is generally difficult for a lot of mainstream backbones.

The SSP models based on decision fusion fuse the respective classification results of spectral and spatial features to predict the final label [67,79,80,157]. As high-level fusion, decision fusion can employ multiple classifiers for different spectral and spatial subnetworks and derives a global decision according to the majority voting rule. But the parameters of spectral and spatial subnetworks may be optimized unevenly, which will limit the discrimination of features to some extent.

The SSP models based on feature fusion integrate spectral and spatial features in different ways, e.g., concatenation and addition, before classification [77,78,80,90,111,119,131,153,161,182,186]. Addition was exploited to aggregate the spectral and spatial features in different modes and keep the consistency of shapes. However, in practice, concatenation was utilized widely because it retained the original structures of features. Moreover, individual weights were assigned to feature neurons in the classification space, which can consider more complex spectral variations and spatial structures. Thus, the SSP models based on feature fusion do better in elevating the discrimination of spectral–spatial features.

##### 3D CNN-Based Models

Nevertheless, the associations between spectral and spatial features are still not considered sufficiently by previous models. The fourth, i.e., 3D CNN based models, were designed to excavate spectral–spatial features from HSI cubes [72,73,81,82,83,84,85,98,102,110,112,114,125,132,133,151,158,160,162,178,187,189,190,191,197]. A 3D convolutional kernel contains both spectral and spatial dimensions. It possesses the advantages of 1D and 2D kernels and reinforces the interactions between spectral and spatial features. During backpropagation, the optimization of kernels takes the gradients in both spectral and spatial dimensions into consideration. Therefore, 3D CNN-based models have been the most appropriate choice for extracting the discriminating spectral–spatial features.

#### 2.3.2. Layouts of Spectral and Spatial Attention Modules

##### Embedding into Separate Subnetworks (ESS)

It is essential for most of models based on deep learning to enhance salient spectral bands and relevant spatial areas during feature extraction. The layouts of spectral and spatial attention modules also have different influences on developing the benefits of spectral and spatial features and promoting the discrimination of features. For the Seq-EA, Seq-AE, and SSP models, both kinds of attention modules are generally embedded into separate subnetworks to emphasize different types of key information [119,161,162,167,171,172,178,182].

##### Different Sequences in 3D CNNs

For 3D CNN-based models, there are two layouts of spectral and spatial attention modules which can be applied. The first layout is that the spectral attention module is placed ahead of the spatial attention module (3D-spe-spa) [164,173,179,184,185,190]. In this way, spectral–spatial feature extraction and subsequent spatial attention will be affected more by salient spectral bands. The second scheme moves the spatial attention module to the front (3D-spa-spe) [95,150,171,187,188,189,198,200], which results in relevant spatial areas playing the main role in feature extraction and the selection of salient spectral bands. A comparison among many related articles in the literature [95,150,162] shows that the second layout is generally more effective than the first one. The most likely reason is that spatial attention highlights relevant areas and suppresses interfering areas, which enables spectral attention to focus on the individuality of the center pixel and exclude the irrelevant representation of interfering pixels. Consequently, the discrimination of spectral–spatial features is improved further.

### 2.4. Enhancing Discrimination of Features in Classification

Feature extraction plays an important role in HSI classification. In previous sections, the crucial points may influence the extraction of discriminating features are summarized. But it is necessary to pay attention to feature optimization in classification parts which can be traditional classifiers [42,43,44,45,46,47,48,49,50,70] and neural network-based classifiers [60,72,73,74,75,76,77,78,79,80,81,82,83,84,85,86]. Traditional classifiers generally measure the distances between features and divide them into different categories. Neural network-based classifiers first transform features into one or multiple classification spaces and then deduce classification scores with softmax or sigmoid activation functions. There are benefits from different loss functions [124,125,126,127,128,129,130,131,132,133,134,135]; the parameters of whole model can be optimized toward the directions of appropriate feature distances and smaller errors, which cannot be realized by traditional classifiers. Thus, the discrimination of features will be enhanced for classification. In this section, some popular loss functions, which are used to control the feature distance, of neural network-based classifiers are illustrated.

Feature distance can be interpreted as intra-class distance and inter-class distance. Neural network-based classifiers adopt generally cross-entropy loss at the last classification layer to evaluate the difference between true label and predicted probability. During optimization, it is difficult for cross-entropy loss to influence feature distances directly. Other effective loss functions, including contrastive loss [120], center loss [121], triple loss [122], and focal loss [123], which exploit different measurements to regulate the feature distances between classes were proposed.

Contrastive loss is the error between deep features of inputs. It aims to ensure that similar inputs cause an identical outcome, and vice versa, which can increase the inter-class distances between classes [124,125]. Center loss supposes that there is a center point for each class and features of each sample should approach the corresponding point as closely as possible during optimization. It was usually installed before the last fully-connected layer to refine the intra-class distances of features [126,127]. To integrate both the advantages of contrastive loss and center loss, triple loss was designed. By receiving two positive samples and a negative sample, triple loss can shrink the feature distribution of each class and expand the inter-class distance [128,129,130]. To avoid the issue that the overall feature distribution was squeezed excessively, an additional margin parameter was added to control the distances between positive and negative samples.

Even though the three loss functions can promote the discrimination of features, the problem of limited samples often causes insufficient optimization. To stabilize the effectiveness of the functions, focal loss improved cross-entropy loss to adjust the optimization weights of different classes [131,132,133,134,135]. The gradients of hard classes with small classification scores will be assigned larger weights during backpropagation. On the contrary, the weights of classes having more samples will be weakened relatively. Hence, the discrimination of features and classification performances will be improved further [217,218,219]. However, the classes with less samples may not be the hard classes due to the complicated spectral characteristics and spatial structures of HSIs, which will reduce the effectiveness of weighted optimization. In general, the usage of these loss functions should take the architectures of the models and properties of the data into full account. Compared with contrastive loss and center loss, triple loss can comprehensively optimize feature distances, but it may spend plenty of time on training. Focal loss may be not suitable for the scenarios whose the numbers of samples of all classes are close. To fit the distributions of hard classes, the combination of focal loss and triple loss can be considered.

## 3. Comparison of Different Techniques

In this section, some classic data sets for HSI classification are first illustrated. Then, the classification performances of some typical methods are reported and analyzed to point out their advantages and disadvantages and potential improvements.

### 3.1. Data Sets for HSI Classification

From the 1990s, more than fifteen data sets from various scenarios have been constructed to assist the research of HSI classification algorithms. Table 1 presents the properties, including collection years, imaging sensors, sizes, spatial resolutions (Spa-Res), spectral resolutions (Spe-Res), wavelength range (WR), number of total bands (*N_b_*), number of available bands (*N*), and number of classes (*N_c_*), of these public data sets.

The Indian Pines (IP) [220], Salinas (SA) [220], and Kennedy Space Center (KSC) [220] data sets were gathered by the Airborne Visible InfraRed Imaging Spectrometer (AVIRIS) sensor. Most classes of the three data sets belong to crops and forests. The Pavia Centre (PC) [220] and Pavia University (PU) [220] data sets were gathered by the Reflective Optics System Imaging Spectrometer (ROSIS) sensor. Buildings and roads are the major classes of these data sets. The Washington DC Mall (WDCM) [221] data set was acquired by the Hydice sensor over Washing ton shopping mall, USA. It contains mainly roads and plants. The Houston2013 (H13) [222] and Houston2018 (H18) [223] data sets were collected by the ITERS Compact Airborne Spectrographic Imager (CASI)-1500 sensor over the University of Houston campus and its neighboring urban areas. The Botswana (BW) [220], Dioni (DN) [224], and Loukia (LK) [224] data sets were obtained by the Hyperion sensor equipped on the Earth Observation (EO)-1 satellite. The latter two belong to the HyRANK hyperspectral benchmark developed by the International Society for Photogrammetry and Remote Sensing (ISPRS). The Xiongan (XA) [225] data set was acquired by the gaofen (GF) series hyperspectral sensor designed by Shanghai Institute of Technique Physics, Chinese Academy of Sciences over Xiongan New Area, China. It is currently the scenario with the largest size. The Wuhan UAV-borne hyperspectral image (WHU-Hi) series data sets were collected by the Headwall Nano-Hyperspec sensor over Longkou (LO) [226], Hanchuan (HC) [226], and Honghu (HH) [226], China, in different years. Both the spectral and spatial resolutions of these are quite high. Crops, such as rice, soybean, and corn, are the main classes of three data sets.

To reduce the influence of other negative factors, including noise, atmospheric refraction, water absorption, etc., these data set have experienced corresponding preprocessing before release. For example, the available bands *N_b_* of some data sets are less than their original bands *N* because the water absorption bands and low signal–noise ratio (SNR) bands were discarded. Therefore, these public data sets can be conveniently employed for research.

### 3.2. Comparison of Classification Performances

In this subsection, the classification performances of different feature-extraction techniques are summarized. The overall accuracy (OA) of some classic methods of the aforementioned eight kinds of techniques, including techniques to extract common spectral features, techniques to represent spectral dependency, techniques to capture salient spectral bands, techniques to extract common spatial features, techniques to deduce relevant spatial areas, different spectral–spatial network architectures, different layouts of attention modules, and loss functions in the classification part, on the corresponding ratios of the training sets are presented in Table 2, Table 3, Table 4, Table 5, Table 6, Table 7, Table 8 and Table 9, separately. The superiority and limitations of these methods are also analyzed in the rightmost columns of those tables. Moreover, some suggestions on how to improve the mainstream deep-learning-based methods in practical application are pointed out in Table 10.

**Figure 3:**
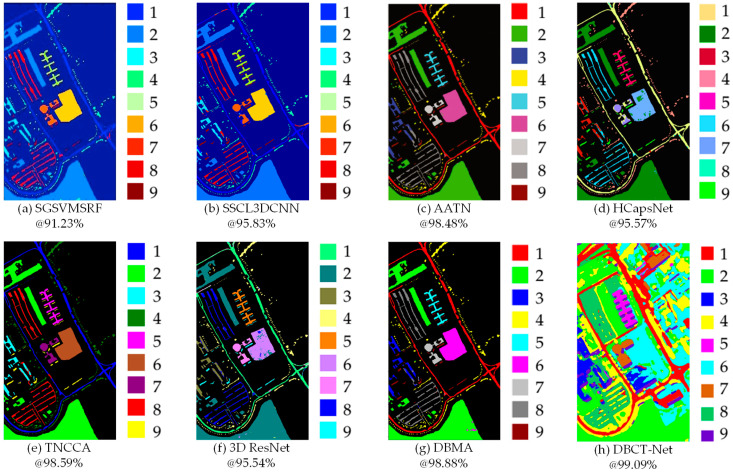
Classification maps of eight typical methods, including SGSVMSRF [23], SSCL3DCNN [90], AATN [157], HCapsNet [98], TNCCA [201], 3D ResNet [187], DBMA [161], and DBCT-Net [131], on the PU [220] data set with 1% labeled samples.

The classification maps of the typical methods of eight techniques to discriminate spectral–spatial features on PU [220] data sets are presented in Figure 3. It can be observed from this figure that the salient bands and relevant areas (Figure 3c,e) play more important roles than the common spectral and spatial features and spectral dependency (Figure 3a,b,d) in improving classification accuracy. Meanwhile, there is less noise and fewer speckles are caused once both key factors are emphasized. Compared with 3D ResNet and DBMA, adopting spectral and spatial attention can obviously elevate the classification performance of backbone networks, such as ResNet [109], DenseNet [115], FCN [92], etc. DBCT-Net is an integration of CNN and transformer, which has a lot of parameters and very high FLOPs. However, the introduction of focal loss enables the model to converge efficiently. Hence, its classification map is quite pure and similar to the ground-truth map. In summary, both attention modules and appropriate loss functions have been the crucial techniques worthy of notice for HSI classification.

## 4. Future Challenges

The above-mentioned techniques have discriminated spectral–spatial features to different extents for classification, but the generalization abilities of these methods still face many challenges. The potential influence factors mainly include the characteristics of hyperspectral data, special generation mechanism of the sample, issue of limited samples, and property of the data set. In this section, the possible challenges that current algorithms may encounter in the future are discussed.

### 4.1. Characteristics of Hyperspectral Data

First, it is well-known that HSIs contain lots of redundant information between bands. Regular dimensionality reduction methods, such as PCA, tend to abandon spectral sequence information. The compressed high-level features of encoder–decoder architectures used for reconstruction may not always be appropriate for classification. To maintain spectral dependency and reduce redundancy, each band and its neighboring bands were integrated as a group and mapped to low-dimensional space [195,200,201,202]. This strategy is similar to the ideology of dilated convolution used to expand the perceptive field. The flexible group size enables the redundancy between bands to be controlled based on the number of bands. Spectral variability is also a unique property of HSIs, which is caused by different environmental conditions. Spectral variability may affect inter-class and intra-class distances. The adaptive estimation methods [187,191] which assign learnable fluctuation coefficients for bands may be valid to deal with spectral variability and recalibrate inter-class similarity.

Second, HSIs consist of hundreds of bands and each band represents the reflectivity of land cover at a certain wavelength, which means that HSIs actually are pseudo-3D data. Existing algorithms, especially for 3D CNN-based algorithms, generally adopt complex 3D convolutional kernels to extract spectral–spatial features. But this may not only obtain redundant spatial features but also increase the number of parameters. Redundant spatial features can interfere with the model’s focus on useful spectral features. Moreover, the spatial resolutions of some data sets are restricted, which will enlarge the inter-class similarity of spatial structures. In this case, some pyramid and multi-scale architectures often make little sense for improving spatial features. Hence, an efficient algorithm for extracting spectral–spatial features is needed. Recently, the combinations of transformer and CNN have been a hotspot in the field of computer vision [201,227,228,229]. Transformer is good at exploring non-local correlations, while CNN has the ability of local modeling. They handle spectral and spatial information adaptively to maximize the respective benefits for HSI classification.

### 4.2. Special Generation Mechanism of Samples

Algorithms based on spectral–spatial features have been the mainstream for HSI classification. As the input of these algorithms, HSI cubes contain the center pixel and its neighborhoods. This special sampling mechanism provides spectral and spatial information for HSI cubes but also introduces irrelevant areas. Features obtained from an HSI cube are the basis to obtain the classification result of the center pixel. Therefore, features should be extracted from the areas related to the center pixel. The validity of centralization ideology has been confirmed by some research studies [150,187,188,190,193]. However, this ideology is still not represented in most current attention modules, including MHSA modules, SE modules, and CBAMs. Another important issue caused by the special sampling mechanism is that the HSI cubes generated from the junction of different classes generally contain a large proportion of same pixels. That means two HSI cubes have similar spatial structures and spectral attributes but may possess different labels. This is disadvantageous to optimize inter-class feature distances. To mitigate this problem, a feasible method is to construct separate optimization for relevant spatial areas and other irrelevant spatial areas, thereby extracting the discriminating spectral–spatial features related to the center pixel for classification.

### 4.3. Issue of Limited Sample

The sample is the bedrock to optimize deep-learning-based algorithms. Sufficient quality and quantitative samples can promote the precise deduction of high-dimensional feature space and convergence of training errors. However, the issue of limited samples, which can be interpreted as a small number of samples and imbalanced numbers of samples between classes, exists commonly in some public data sets, such as IP, PU, DN, LK, etc. Models tend to concentrate excessively on the classes with more samples and extract undiscriminating features, thereby forming skew decision borders. Data augmentation, an available method to relieve the issue of limited samples, is easy to conduct but generally results in slight improvement and additional computational cost. This is because new samples were actually copied from existing samples using operations of clip, rotation, etc. Recently, some popular generative models, including diffusion models [230,231,232,233], Sora models [234,235], and GANs, can be considered to produce effective samples based on the high-level semantic understanding of the original data. The focal loss function [219], which adaptively varies the weights of different classes to guide models to focus on those classes with a small number of samples, is also a good choice to cope with the issue of limited samples without extra training consumption.

### 4.4. Type of Land Cover of Data Set

Among the properties of an HSI data set, the type of land cover, which is related to the imaging scene, may have a large influence on improving the ability of models to represent discriminating features. Spectral signatures of similar kinds of land cover usually contain subtle differences in certain band wavelengths, which facilitates models to extract the crucial features between different classes to some degree. However, the scenes of the present public HSI data sets contain mainly farm, city, mountain, etc. There are various features with very different spectral signatures, such as plants, roads, and water. Therefore, constructing HSI data sets with highly similar types of land cover may be also a feasible solution to improve the discriminating spectral–spatial feature representation and robustness of algorithms in realistic scenes.

## 5. Conclusions

HSI classification is one the most important application of HSI analysis. Because of the redundancy between bands and complicated spatial structures, the effectiveness of shallow features extracted by traditional machine-learning-based methods are generally weak. Recently, algorithms based on deep learning have been the mainstream means to extract discriminating spectral–spatial features for classification. In this article, the important techniques to strengthen the discrimination of features are summarized from the aspects of feature extraction and feature optimization. For the discriminating spectral features, models should pay more attention to spectral dependency and salient spectral bands. Capturing relevant areas is necessary to ensure the effectiveness of spatial features. Different network architectures and combinations of attention modules to obtain discriminating spectral–spatial features are also presented in detail. During feature optimization, the loss functions for controlling feature distance are illustrated. Moreover, the excellence, deficiencies, and potential improvements of typical techniques are analyzed. Finally, the possible challenges which may be helpful for improving future study on the discrimination of spectral–spatial features are pointed out further.

## Figures and Tables

**Table 1:** Details of some classic hyperspectral data sets.

Name	Year	Sensor	Size	Spa-Res (m)	Spe-Res (nm)	WR (nm)	*N_b_/N*	*N_c_*
Indian Pines	1992	AVIRIS	145 × 145	20	10	400–2500	224/200	16
Salinas	1992		512 × 217	3.7			224/204	16
Kennedy Space Center	1996		512 × 614	18			227/176	13
Pavia Centre	2002	ROSIS	1096 × 1096	1.3	4	430–860	115/103	9
Pavia University	2002		610 × 340					9
Washington DC Mall	2013	Hydice	307 × 1280	2	10	400–2400	210/191	7
Houston2013	2013	ITERS CASI-1500	349 × 1905	2.5	4.653	380–1050	144/144	15
Houston2018	2018		601 × 2384	1	13.958		48/48	20
Botswana	2001	Hyperion	1476 × 256	30	10	400–2500	242/145	14
HyRANK-Dioni	2018		250 × 1376				242/176	12
HyRANK-Loukia	2018		249 × 945				242/176	14
Xiongan	2017	GF series	1580 × 3750	0.5	2.4	400–1000	250/250	19
WHU-Hi-Longkou	2018	Headwall Nano-Hyperspec	550 × 400	0.463	2.2	400–1000	270/270	6
WHU-Hi-Hanchuan	2016		1217 × 303	0.109				16
WHU-Hi-Honghu	2017		940 × 475	0.043				22

**Table 2:** Comparison of typical methods to extract common spectral features.

Year	Method	Training Ratios	OAs	Highlights and Limitations
2018	A method using spectral gradient, SVM and spatial RF (SGSVMSRF) [23]	IP—5% PU—2% SA—3%	IP—97.41% PU—98.97% SA—97.85%	~It integrated multi-scale spectral gradient information and predicted with SVM and RF classifiers. ~The generalization ability of this method was limited due to single features, and the test procedure was time-consuming.
2008	Scale-invariant feature transform for spectral matching (SIFT-SM) [36]	Not applicable	WDCM—85.10%	~It extracted SIFT features from spectral curves and classified them with spectral matching and the minimum distance. ~The issue of spectral variability was not considered so that the accuracy of some individual classes was very low.
2019	Spectral-adaptive segmented DBN (SAS-DBN) [61]	10%	H13—98.35% PU—93.15%	~It prepared the spatial and spectral segmentations and separate correlated bands for DBN. ~The classification performance was limited by the number of bands which can influence the complexity of correlated bands.
2017	Hyper-voxel stacked auto-encoder (HVSAE) [69]	10%	IP—90.08% PU—98.98%	~Multi-layer stacked AEs were exploited to represent the HVs based spatial and spectral features. ~The efficiency of method was related to the number of HVs which was generally hard to decide.
2016	1D CNN [72]	IP—22% PU—10% KSC—10%	IP—87.81% PU—92.28% KSC—89.23%	~It utilized the classic 1D CNNs to extracted spectral features from spectra and the logistic regression was used for classification. ~This model considered spectral information only, thus the classification accuracy was limited even with more samples.

**Table 3:** Comparison of typical methods to represent spectral dependency.

Year	Method	Training Ratios	OAs	Highlights and Limitations
2017	Deep recurrent neural network (DRNN) [64]	PU—10% H13—20% IP—7%	PU—88.35% H13—89.85% IP—88.63%	~It explored the intrinsic sequential structures of spectra with the help of a parametric rectified tanh activation function. ~Gated recurrent unit not good at modeling long-term dependency.
2024	Hybrid CNN Bi-LSTM [65]	30%	IP—99.83% PU—97.70% SA—97.40%	~The extraction of spatial and spectral features was seen as a sequence learning problem and the Bi-LSTMs were built. ~Spectral dependency has been abandoned by the band reduction before LSTM modules.
2017	Convolutional recurrent neural network (CRNN) [89]	10%	H13—97.08% IP—96.61%	~Convolutional layers were introduced to extract the middle-level and local invariant features before recurrent layers. ~More iterations were need to realize the convergence of the network.
2020	Spatial–spectral ConvLSTM 3D neural network (SSCL3DNN) [90]	IP—10% SA—1% PU—1%	IP—98.79% SA—99.29% PU—97.10%	~Each band of the local patch of spectrum was input to process the long-range spectral dependencies. ~Overfitting may occur when the number of samples is less.

**Table 4:** Comparison of typical methods to capture salient spectral bands.

Year	Method	Training Ratios	OAs	Highlights and Limitations
2019	Semi-supervised 3D CNN with adaptive dimensionality reduction (3D CNN-ADR) [81]	10%	IP—97.89% PU—98.45% SA—98.29%	~The informative spectral bands were selected adaptively to refine spectral and spatial features. ~The differences between salient bands of classes were ignored.
2021	2D CNN with compact band weighting module (2D CNN-CBW) [149]	IP—10% PU—1% SA—1%	IP—97.05% PU—95.83% SA—95.82%	~A lightweight model integrating 2D convolution and SE attention. ~2D convolutional kernels cannot characterize complicated local spectral features.
2021	Residual spectral attention network (RSeAN) [162]	IP—20% PU—10% KSC—20%	IP—95.67% PU—99.20% KSC—96.79%	~It emphasized salient bands of spatial features with the channel attention of CBAM to recalibrate spectral features. ~The 2D CNN backbone was not appropriate for HSI cubes/patches.
2023	Efficient channel attentional feature fusion dense network (CA-FFDN) [154]	IP—20% PU—10% KSC—20%	IP—99.51% PU—99.91% KSC—99.89%	~The adaptive selection of kernel size was utilized to improve the accuracy of key bands. ~The computation burden was heavy due to densely connection.
2022	Aggregated-attention transformation network (AATN) [157]	1%	PU—98.48% LO—99.68% H18—88.59%	~Different pooling methods were applied and aggregated with raw spectra to provide more references for spectral attention. ~The fixed shapes of transformations restricted the flexibility of the model.

**Table 5:** Comparison of typical methods to extract common spatial features.

Year	Method	Training Ratios	OAs	Highlights and Limitations
2014	Sparse representation with extended LBP (SS-ELBP) [29]	Not applicable	IP—91.07%	~The spatial LBP features related to the center pixel were explored. ~The shallow features still suppressed classification accuracy.
2011	3D fused Gabor-wavelet-based SVM approach (3D FG-SVM) [33]	5%	IP—96.04% KSC—95.36%	~Different frequencies and orientations of Gabor wavelets were proposed to sense both spectral and spectral variances. ~More shallow features were required for the classifier to fit data distribution.
2008	A method using SVMs and extended morphological profile (SVM-EMP) [34]	PU—10% PC—5%	PU—83.53% PC—99.69%	~The spatial relationships between pixels in several primary components of data were constructed. ~The redundant morphological profiles may be produced.
2016	2D CNN [72]	IP—22% PU—10% KSC—10%	IP—89.99% PU—94.04% KSC—94.11%	~A 2D CNN was deployed to extract deep spatial features. ~The crucial spectral information was not focused on.
2023	GCN using adaptive neighborhood Laplacian matrix (AN-GCN) [91]	IP—7% H13—20%	IP—88.51% H13—97.88%	~It used the statistics information of neighborhoods to promote the representation ability of a deep graph network. ~This model was sensitive to the number of training samples.
2021	Hybrid capsule network (HCapsNet) [98]	IP—5% PU—10% SA—1%	IP—97.34% PU—98.54% SA—90.01%	~Capsule units excavated the hierarchy between parts of spectral and spatial entities. ~Both the time and the computation cost of the training procedure were heavy.
2020	Structure aware GAN (SA-GAN) [106]	IP—6% H13—20% H18—7% PU—10%	IP—91.95% H13—88.18% H18—75.62% PU—99.82%	~Structure-aware fake samples with high quality were generated for classification. ~The issue of mode collapse was very common during the training process of the GAN model.
2018	Spectral–spatial residual network (SSRN) [110]	IP—20% PU—10% KSC—20%	IP—99.19% PU—99.79% KSC—99.61%	~The introduction of the residual technique promotes the optimization of the spectral–spatial network. ~Larger 3D filters cost a long time on feature extraction.
2021	Multi-scale densely-connected convolutional network (MS-DenseNet) [117]	WDCM—3% PU—1% H13—1.5% SA—1.5% IP—5%	WDCM—97.74% PU—99.32% H13—95.91% SA—99.50% IP—97.66%	~The multi-scale spectral and spatial information in multi-scale samples and different layers were employed to elevate classification accuracy. ~Spectral correlations may be neglected due to the fact that the inputs were compressed by PCA.

**Table 6:** Comparison of typical methods to deduce relevant spatial areas.

Year	Method	Training Ratios	OAs	Highlights and Limitations
2022	Hybrid-convolution and hybrid-resolution network with double attention (H^2^A^2^Net) [171]	PU—0.5% SA—0.5% KSC—3% H13—3%	PU—97.71% SA—97.79% KSC—96.29% H13—96.69%	~A double attention module was applied to highlight the useful information of features extracted with hybrid CNN. ~The attention module played a role in final feature selection only, which could not improve feature extraction.
2021	Residual spatial attention network (RSaAN) [162]	IP—20% PU—10% KSC—20%	IP—89.68% PU—99.30% KSC—97.52%	~It emphasized important areas with the spatial attention of CBAM to recalibrate spectral features. ~The 2D CNN backbone was not appropriate for HSI cubes/patches.
2022	3D fully convolutional neural network (3D FCNN) [178]	IP—50% PC—10% PU—10% BW—10% SA—10%	IP—99.25% PC—99.63% PU—99.60% BW—97.02% SA—96.97%	~Bottle attention was introduced to reduce redundant information and an FCN was adopted to avoid the loss of data. ~Lots of samples were need for optimization and the training time was also very long.
2020	Spectral–spatial attention network (SSAN) [95]	IP—10% PU—2% SA—2%	IP—95.49% PU—98.02% SA—96.81%	~A self-attention module was embedded into the spectral–spatial network to discriminate features. ~Both training and test procedures were inefficient due to complex matrix operations of self-attention.
2024	Hierarchical attention transformer (HAT) [181]	LO—0.1% SA—0.5% LK—1.5% BW—6%	LO—99.89% SA—99.56% LK—91.75% BW—99.59%	~A hierarchical self-attention module was used to improve high-level feature representation and reduce computation pressure. ~Spectral dependency may be lost during PCA.
2022	A spectral-similarity-based spatial attention module (S^3^AM) [187]	IP—5% PU—2% LK—5% XA—1%	IP—93.31% PU—97.99% LK—94.25% XA—88.95%	~It deduced relevant areas with weighted Euclidean and cosine distances and scalable Gaussian activation. ~The common attention modes which have a weak link to the center pixel were not explored.
2021	Center attention network (CAN) [189]	IP—10% PU—2% SA—2%	IP—98.10% PU—98.97% SA—98.18%	~The ideology of centralization was introduced into the self-attention module to capture the global correlations related to the center pixel. ~The computation burden of the training procedure was heavy and common attention modes were also neglected.
2024	Center attention transformer with stratified spatial–spectral token (CAT-SSST) [193]	IP—7% PU—10% H13—20%	IP—93.69% PU—99.05% H13—93.21%	~Super-pixel region sampling mechanism was designed to generate purer HSI cubes for improving CAT structure. ~Preparing samples and the training of models required lots of time.
2022	Spectral transformer patch-wise (SFP) [200]	IP—7% PU—10% H13—20%	IP—81.76% PU—91.07% H13—88.01%	~It introduced the first transformer to HSI classification and proposed pixel-wise and patch-wise models. ~Both time and storage complexities were high.
2023	Spatial–spectral 1DSwin Transformer (SS1DST) [206]	IP—7% PU—10% H13—20% SA—2%	IP—89.66% PU—93.04 H13—90.46% SA—95.45%	~1D Swin transformers were applied to model local and hierarchical spatial–spectral relationships. ~The relevant areas may be destroyed by different shifted windows during attention inference.
2024	A transformer network with a CNN-enhanced cross-attention (TNCCA) [201]	1%	H13—90.72% PU—98.59%	~Transformer was exploited to extract deep-level features and fuse different-scale information. ~The setting of multi-scale increased the computational complexity.

**Table 7:** Comparison of typical methods with different architectures.

Year	Method	Training Ratios	OAs	Highlights and Limitations
2020	Spectral–spatial convolutional network (SSN) [95]	IP—10% PU—2% SA—2%	IP—94.65% PU—97.37% SA—96.23%	~The spectral and spatial modules were connected in sequence to extract spectral–spatial features. ~3D convolutional kernels made it hard to reach convergence.
2018	Non-local spatial sequential RNN (NLSS-RNN) [63]	IP—10% PU—9% SA—10%	IP—98.75% PU—99.77% SA—97.23%	~The local spatial features and relationships between pixels were both considered to represent rich spectral features. ~Spatial features were obtained with hand-crafted descriptors.
2022	A deep CNN with local similarity projection Gabor filtering (DCNN-LSPGF) [77]	20%	IP—98.91% PU—97.25% SA—97.25%	~Both spectral and spatial information processed by Gabor filtering were fused before the feature extractor. ~Samples’ generation could be optimized with the network.
2019	Spectral–spatial LSTMs (SSLSTMs) [66]	10%	IP—95.00% PU—98.48% KSC—97.89%	~The respective classification results of spectral and spatial LSTM modules were fused to obtain the final predictions. ~The rows of spatial input were split, which lost local relationships.
2023	Dual-stream fusion network (DSFN) [182]	IP—10% PU—5% SA—10% KSC—15%	IP—98.77% PU—99.83% SA—99.67% KSC—98.90%	~A weighted fusion between both spectral and spatial features was conducted before classification. ~Some local structures of spectral and spatial features may be damaged before fusion by fully-connected layers.
2022	3D ResNet [187]	IP—5% PU—2% LK—5% XA—1%	IP—86.54% PU—95.54% LK—80.55% XA—83.99%	~It exploited 3D residual convolution only to extract spectral–spatial features from HSI cubes/patches. ~It was difficult to confirm the proper kernel sizes of spectral and spatial modules.

**Table 8:** Comparison of typical attention-based methods with different layouts.

Year	Method	Training Ratios	OAs	Highlights and Limitations
2019	Double-branch multi-attention (DBMA) network [161]	IP—5% PU—1% SA—1%	IP—98.19% PU—98.88% SA—98.04%	~The channel attention and spatial attention were embedded into spectral and spatial dense modules, respectively. ~The dense connections and attention blocks resulted in additional time consumption when tested.
2021	Residual spectral–spatial attention network (RSSAN) [162]	IP—20% PU—10% KSC—20%	IP—99.46% PU—99.89% KSC—99.74%	~It emphasized salient bands and important areas to obtain discriminating spectral–spatial features. ~It was limited by the 2D CNN backbone which was not appropriate for HSI cubes/patches.
2022	Spatial attention guided residual attention network (SpaAG-RAN) [150]	IP—15% PU—5% BW—15%	IP—98.34% PU—99.04% KSC—99.74%	~The spatial attention module promoted the work of the spectral attention module and feature extraction. ~Spatial attention may derivate due to a single distance metric.

**Table 9:** Comparison of typical methods with different loss functions.

Year	Method	Training Ratios	OAs	Highlights and Limitations
2022	Cross-domain CNN with contrastive loss (CDCNN-C) [125]	2%	IP—96.40%	~Contrastive learning between real samples and unlabeled samples was performed to improve accuracy. ~The time complexity of the cross-domain CNN was very high.
2024	Spectral–spatial residual network with center-boundary metric loss (SSRN-CBML) [126]	IP—3% PC—1% PU—1% SA—1%	IP—93.58% PC—98.93% PU—99.73% SA—94.56%	~The center loss was used to enhance the discrimination of features. ~The algorithm contained feature extraction and classification parts, which was not an end-to-end model.
2022	Triplet-watershed network (TWN) [130]	10%	IP—99.57% PU—99.98% KSC—99.72%	~The implicit connectivity patterns of data sets were found by a watershed classifier. ~More complex feature extractors were required for the classifier.
2024	Double branch convolution-transformer network (DBCTNet) [131]	PU—1% H13—5% LO—1%	PU—99.09% H13—98.60% LO—98.16%	~The focal loss was deployed to improve the difference between easy and hard classes. ~The architecture of double-branch and many SA modules limited the portability of the model jointly.

**Table 10:** Potential improvements of some mainstream deep-learning-based methods.

Techniques	Potential Improvements
RNNs, LSTMs, ConvRNNs, ConvLSTMs	Construct bi-directional RNNs or LSTMs or stack them to enhance sequential spectral and spatial features. Simplify the structure used to explore sequential dependency to reduce the computational complexity of models. Introduce an attention mechanism to help the model to focus on different sequences.
CNN-based architectures	Replace regular convolution with depth-wise convolution or separate convolution to reduce parameters and training costs, especially for 3D CNN-based methods. Integrate CNN with an attention mechanism to select useful features adaptively. Adopt multiple kernel sizes or dilation rates to improve the receptive field of kernels and represent different scales of features.
Transformer architectures	Combine transformers and other models which can extract local correlations, such as CNNs, to represent comprehensive features. Reduce bands or simplify architectures to decrease both the time and computational complexity of MHSA modules. Improve MHSA modules to concentrate on the global dependency related to relevant pixels, which can take full use of crucial information. Features of different encoders can be considered for feature fusion or decision fusion.
Spectral attention modules	The information related to the center pixel, especially for the difference between the center pixel and its neighborhoods, are worth highlighting when generating spectral attention. Adopt a lightweight structure to make the spectral attention module efficient against numerous bands of HSIs.
Spatial attention modules	Ensure that the center pixel is the core during the inference of spatial attention. Increase types of information, such as contextual information and spectral features, for reference to improve the accuracy of attention.

## Data Availability

Not applicable.
